# Poison of Serpents

**Published:** 1881-12

**Authors:** 


Recent observations by M. Gautier (communicated to the Paris Académie de Médecine) afford reason for believing that the poison of serpents differs from human saliva in the intensity of its effects rather than in essential nature, so that the fears with which a human bite is often regarded may not be wholly unreasonable. M. Gautier took some twenty grammes of human saliva, and, after lixiviating and purifying, obtained a substance which, injected in the form of solution under the skin of a bird, had remarkable toxical effects. Almost immediately the bird was seized with trembling. It staggered and fell to the ground in a state of coma and complete stupor, terminated by death in half an hour or and hour, according to the dose injected and the vigor of the animal. The phenomena resembled fully those produced by the bite of a venomous serpent. The poisonous matter of the saliva is thought to be an alkaloid similar to the cadaveric poisons called *ptomaines*, which MM. Brourdel and Boutmy have isolated. Like them, it produces Prussian blue when mixed with ferrocyanide of potassium. The facts stated throw some light on the question of virulent maladies. The present case, it is pointed out, is not that of a true virus; for at high temperatures a virus is destroyed, but when the salavary alkaloid is heated to more than 100 degrees its poisonous property is not affected. M. Gautier studied comparatively the poison of the cobra (one of the most formidable Indian serpents). This, injected in a dose of one milligramme in a quarter of a cubic centimetre of water under the skin of a small bird, such as a chaffinch or a sparrow, kills it in five to twelve minutes. One observes torpor and coma, then a period of excitation, with convulsions and tetanic contraction. In connection with the subject, a correspondent of *La Nature* calls attention to a passage of Rabelais, in which the poisonous nature of human saliva is recognized.

